# Laparoscopic S7 hepatectomy for hepatic mucinous neoplasm: a case report and literature review

**DOI:** 10.1186/s12876-021-02059-y

**Published:** 2021-12-20

**Authors:** Yongming Zhang, Yong Wei, Yu Cheng, Fang Liu, Haitao Wang, Lili Jing

**Affiliations:** 1 Department of Hepatobiliary, Pancreatic and Spleen Surgery, Yantai Affiliated Hospital of Binzhou Medical University, Yantai, 264100 Shandong People’s Republic of China; 2 Quality Management Office, Yantai Affiliated Hospital of Binzhou Medical University, Yantai, 264100 Shandong People’s Republic of China; 3 Department of Pathology, Yantai Affiliated Hospital of Binzhou Medical University, Yantai, 264100 Shandong People’s Republic of China

**Keywords:** Laparoscopy, Liver tumor, Mucinous cystic neoplasm

## Abstract

**Background:**

Mucinous cystic neoplasm of the Liver is rare tumors with malignant potential that occur in the biliary epithelium. Because of its rare presentation, it is often misdiagnosed before surgery.

**Case presentation:**

A 63-year-old female patient presented with intermittent upper abdominal pain for three months. Laparoscopic hepatectomy of Segment 7 was conducted based on the preoperative diagnosis of space-occupying lesion in the right lobe of the liver. Postoperative pathology showed a low-grade mucinous cystic neoplasm in the right posterior lobe of the liver. The preoperative CA19-9 level was significantly increased while the postoperative CA19-9 returned to the normal range.

**Conclusions:**

The diagnosis of mucinous cystic neoplasm of the liver is closely related to the thickening of the cystic wall or the increase of CA19-9, which has great significance and deserves clinical attention.

## Background

Mucinous cystic neoplasm (MCN) of the liver was officially proposed in the fourth edition of the World Health Organization’s (WHO’s) new classification of digestive system tumors in 2010 [1], which was originally called biliary cystadenoma or cystadenocarcinoma [2]. WHO(2019) Classification of Tumors of the Digestive System clearly defines MCN as a cystic tumor composed of cubic or columnar epithelium and subepithelial ovariole stroma. The cystic cavity is generally not connected to the bile duct system. Based on the atypical epithelium and structure, non-invasive MCN is classified as low grade and high grade [3]. MCN is an extremely rare tumor that occurs primarily in middle-aged women. It originates from the biliary epithelium with malignant potential. Because of the lack of clinical reports on this condition exist, it is easily misdiagnosed in the preoperative period. We report one case admitted to our department in May 2020 with a subsequent review of the literature.

## Case presentation

**Fig. 1:**
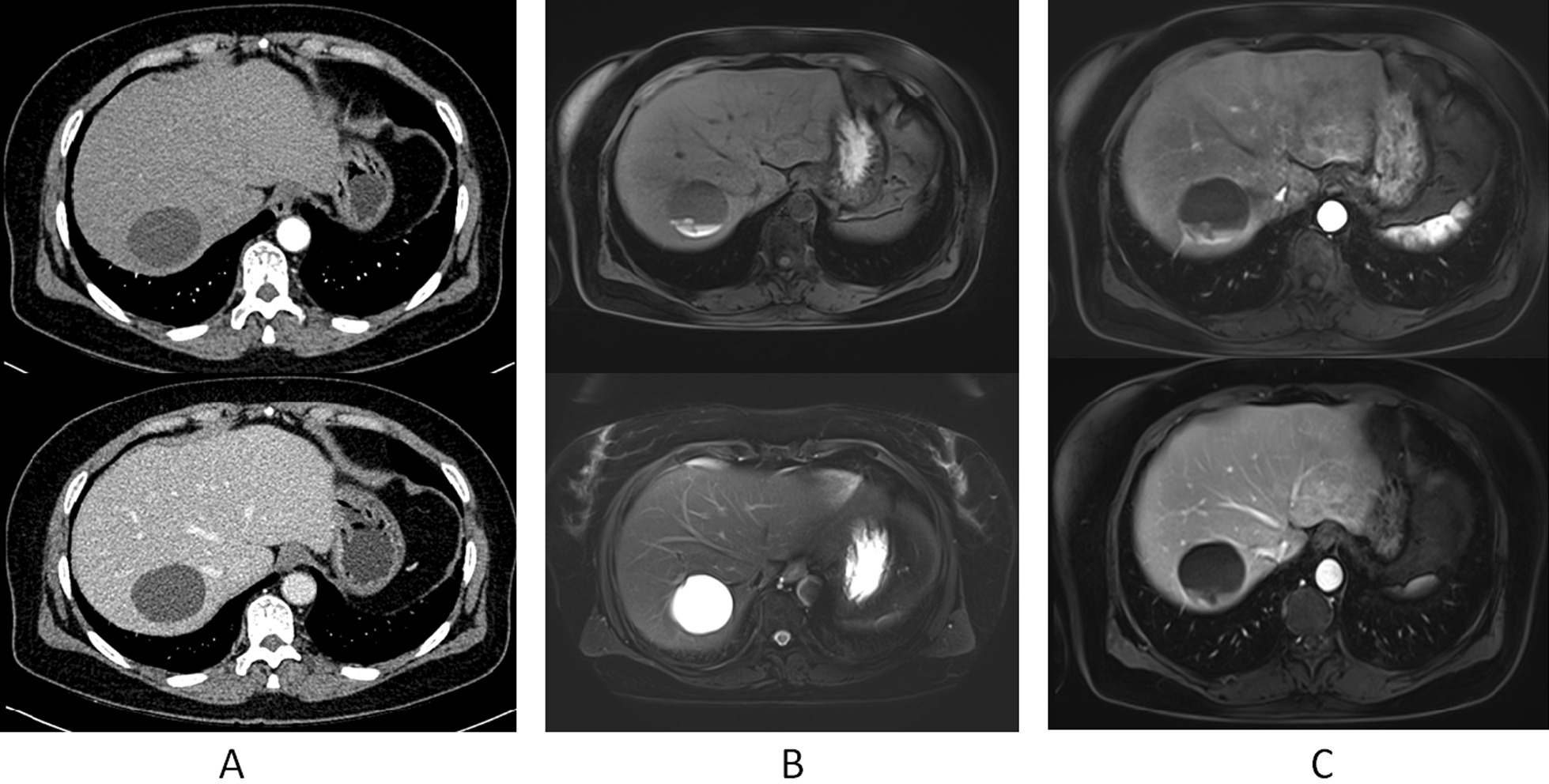
Preoperative imaging examination. **A** Upper abdominal enhanced computed tomography. **B** Hepatobiliary T1, T2 magnetic resonance imaging. **C** Hepatobiliary enhanced MRI

**Fig. 2:**
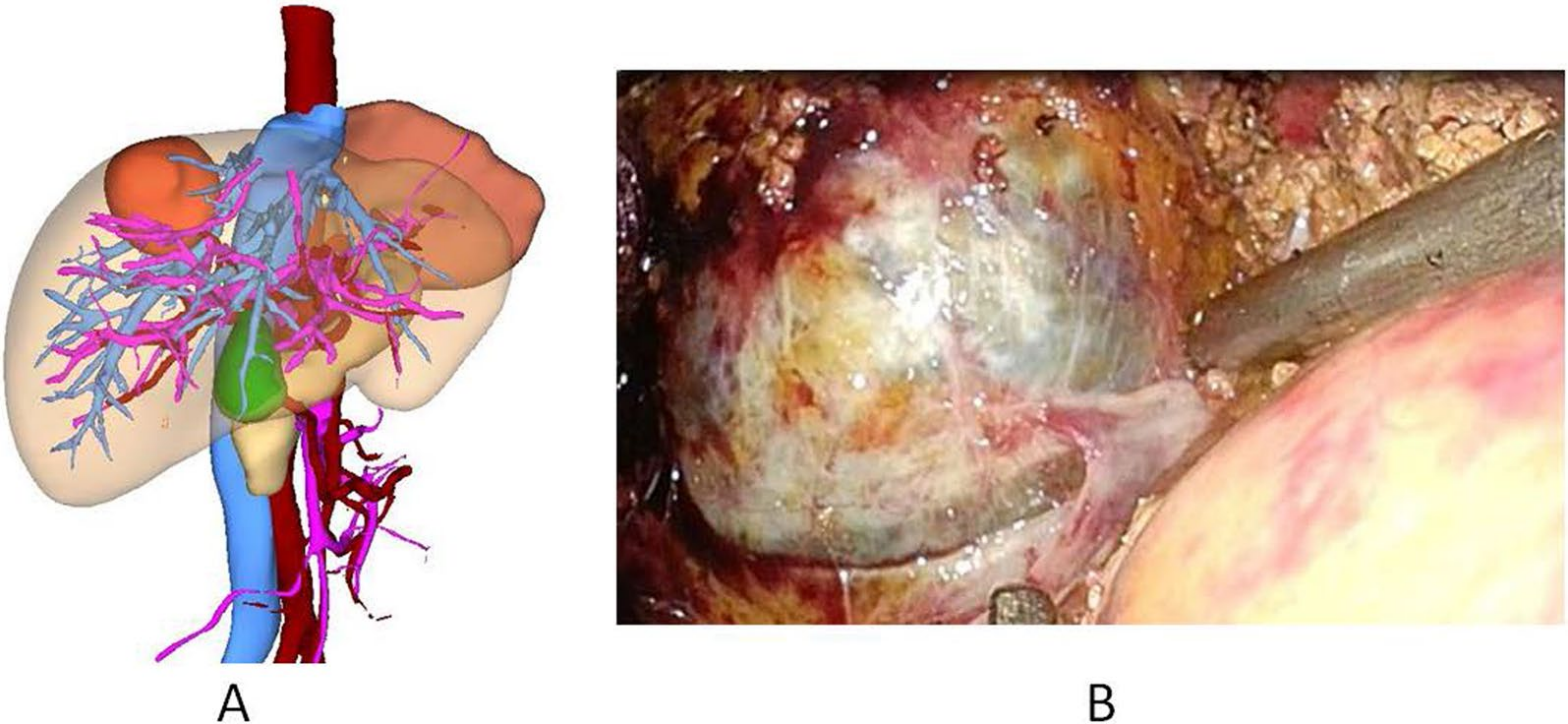
Three-dimensional reconstruction and Intraoperative photographs. **A** Three-dimensional reconstruction. **B** Intraoperative tumor location and relationship with the right hepatic vein

A 63-year-old female patient was admitted to our hospital with intermittent epigastric abdominal pain for the past three months. Results of the physical examination on admission indicated no icteric sclera. The abdomen was soft with no palpable abdominal mass. The patient had experienced a weight loss of about five kg in the past two months and had no history of hepatitis B or C. She had been exposed to dogs and sheep and denied any history of exposure to infected cases from the epidemic area. After admission, CA19-9 was measured and was 796.20 U/mL. No serological examination for echinococcosis was performed because of the limited conditions of our hospital. Test results from pelvic ultrasound indicated menopausal uterus and uterine fibroids. Test results from gastroscopy showed chronic non atrophic gastritis. Colonoscopy revealed multiple polyps in the large intestine (basically removed); intestinal histopathology (cecum, biopsy) showed severe chronic inflammation of the mucosa and adenomatous hyperplasia of the individual glands. Contrast-enhanced computed tomography of the upper abdomen (Fig. 1A) revealed a more homogeneous thickening of the gastric wall in the antrum. Round unenhanced low-density foci with a diameter of 4.6 cm was seen in the S7 segment of the liver. Nodular calcifications were also observed. No significant dilatation was noted in the intrahepatic and extrahepatic bile ducts. The size and shape of the gallbladder were normal, the wall was not thick, and no significant abnormal density was observed in the cavity. The pancreas, spleen, and adrenal glands showed no significant abnormalities. Test results from computed tomography indicated liver cyst and intrahepatic calcifications. Contrast-enhanced magnetic resonance imaging of the liver and gallbladder (Fig. 1B, C) revealed a normal size and shape of the liver and proportion of each lobe, and the intrahepatic and extrahepatic bile ducts and flow vessels ran naturally. A long T1 and long T2 cystic signal with a diameter of about 4.7 cm was observed in the right lobe of the liver, with liquid level, short T1 high signal intensity in the lower layer, high signal intensity on diffusion-weighted imaging sequence, and enhancement of the cyst wall on the enhanced scan. Test results from magnetic resonance imaging showed that the space-occupying lesion of the right lobe of the liver had been considered to be more likely a hepatic hydatid cyst. Preoperatively, three-dimensional reconstruction demonstrated the location of the tumor and its relationship with the surrounding vessels (Fig. 2A).

The patient underwent laparoscopic S7 segmentectomy. Intraoperative findings showed that the tumor was located at the S7 segment of the liver and was about 5 × 4 cm in size and partially protruding from the surface of the liver, with an intact capsule and clear boundary with normal liver tissue. The tumor compressed the right hepatic vein and its tributaries and densely adhered to the right hepatic vein (Fig. 2B). On postoperative pathology, a mass was observed immediately adjacent to the liver capsule, with a volume of 5 × 5 × 4.5 cm. The section surface showed a brown turbid fluid, a smooth inner wall, and greyish red, greyish yellow, and soft section surfaces of other liver tissues. The pathological section showed a low-grade mucinous cystic neoplasm (volume 5 × 5 × 4.5 cm) in the S7 segment of the liver, with steatosis in the surrounding hepatic tissue area, chronic inflammatory cell infiltration in the portal area, and no tumor cell involvement in the margin of the liver resection.

**Fig. 3:**
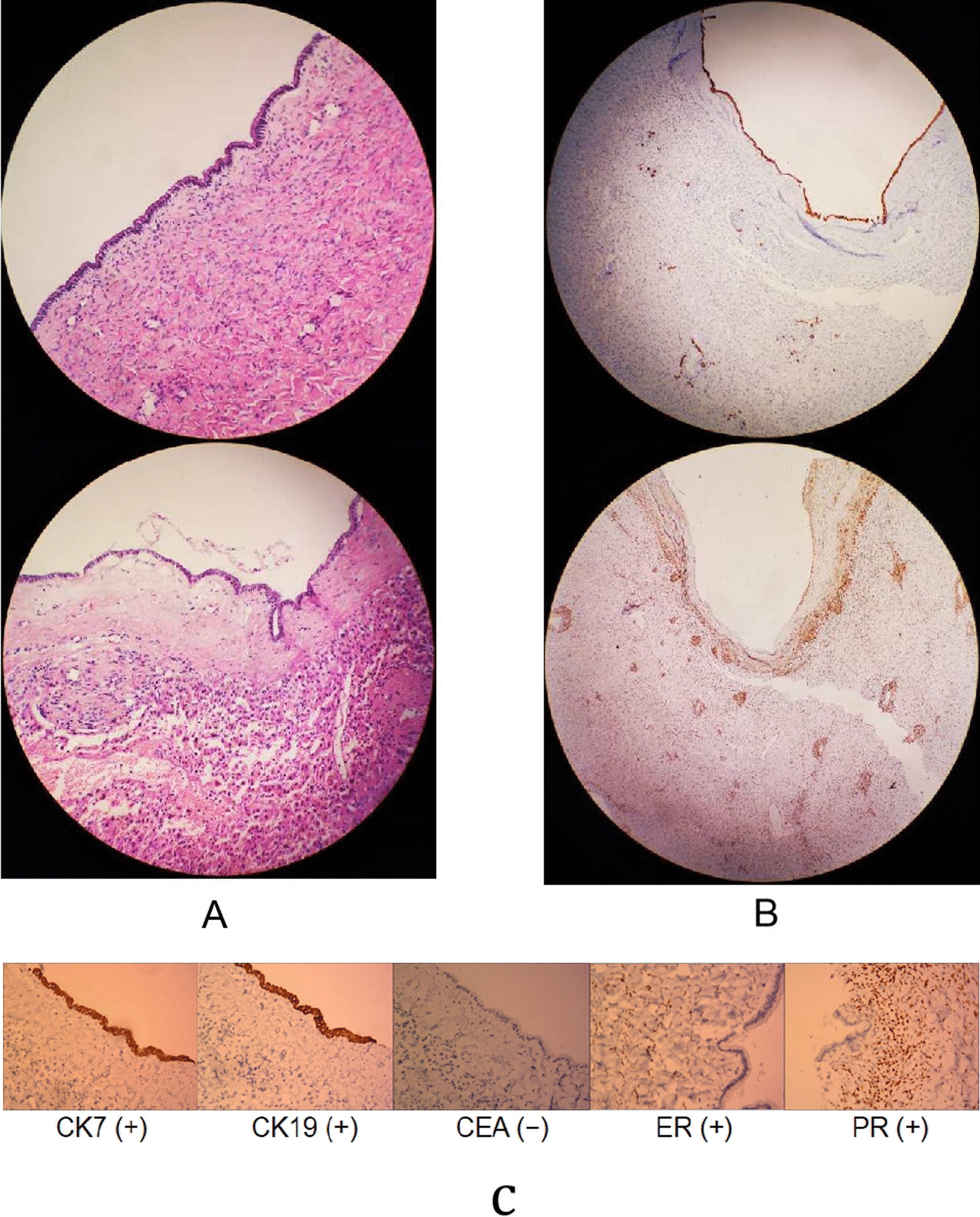
Pathological examination results. **A** Pathological examination results of liver tumors in the patient (hematoxylin and eosin [HE] stains ×100). **B** Pathological examination results of liver tumors in the patient (hematoxylin and eosin [HE] stains ×200). **C** Immunohistochemistry showed CK7 (+), CK19 (+), CEA (−) in tumor cells, and ER (+), PR (+) in stromal cells

Immunohistochemistry demonstrated tumour cells CK7 (+), CK19 (+), and CEA (−); stromal cells ER (+), PR (+), α-inhibin (a small amount +), vimentin (+), desmin (+), and actin (+) (Fig. 3 A–C). This study was approved by the Ethics Committee of Yantai Affiliated Hospital of Binzhou Medical University.

## Discussion and conclusions

Mucinous tumors can occur in the ovary, pancreas, and retroperitoneum, but are rare in the liver. Therefore, close attention should be paid to cases with a preoperative imaging diagnosis that is solid, no other mass, and a significantly increased level of CA19-9 level. The preoperative CA19-9 level, in this case,was significantly increased, which may be related to cholestasis and tumor compression of the bile duct. MCN contains ovarian-like interstitial receptors expressing progesterone and estrogen, and most (about 75%) liver MCNs are located in the left lobe of the liver [4]. Compared with ovarian MCN, liver and pancreatic MCN have different steroid production characteristics, especially in androgen synthesis. Androgens seem to be the key to the occurrence of hepatopancreatic MCN. [5]. Many women have been diagnosed with MCN. Studies [6] have shown that female MCN usually occurs in perimenopausal women (average age 50 years), which further supports that the occurrence of MCN is related to the hormonal environment. The diagnosis of hepatic mucinous tumors is critical. Most patients with MCN have no obvious subjective symptoms. Some patients may experience abdominal discomfort and pain because of large masses, or they may present with a fever resulting from jaundice or inflammatory reactions due to compression of the bile duct by the masses. Cases of acute epigastric pain attacks have also been reported [7]. Some studies [8] have found that preoperative MDT is of great value in differentiating hepatic cysts from MCN. Kunovsky et al. [9] reported a case in which MCN caused dilatation of the left hepatic duct and communicated with the bile duct. In clinical practice, MCN should be differentiated from the hepatic cyst, hepatic echinococcosis, papillary tumor of the bile duct, and liver abscess. The WHO recently defined hepatic mucinous tumor and intraductal papillary tumor of the bile duct from gross and histological aspects, respectively. Intraductal papillary tumor communicates with the intrahepatic bile duct, but there is no ovarian-like stroma.

Mucinous tumors of the liver are often misdiagnosed and treated improperly, but the prognosis is good after complete resection [10]. At present, surgical options include traditional open resection and laparoscopic minimally invasive resection. Laparoscopic minimally invasive surgery has more obvious advantages than traditional surgery for the treatment of hepatic mucinous tumors. As early as 2016, a study reported [11] the case of a patient who underwent laparoscopic resection of the hepatic mucinous tumor. The patient recovered quickly after laparoscopic resection of the hepatic mucinous tumor and was discharged one week after the operation. The postoperative measurement revealed that the patient’s CA19-9 level returned to the normal range (13.26 U/mL). The prognosis of patients with MCN without associated invasive carcinoma is good, but these patients have the potential for malignant transformation. Some studies [12] have found that epithelial mucinous transformation is closely related to the malignant transformation of MCN, but there is a risk of progression to malignant tumors only when the mucinous epithelium is abundant. The prognosis of associated invasive carcinoma derived from MCN can be predicted, but the current study suggests that it is better than hepatobiliary cell carcinoma [1].

MCN is a rare but very challenging disease. Making a definitive diagnosis before the operation is often difficult. CA19-9 serum levels are elevated in the minority of cases with mucinous cystic liver neoplasms. If not diagnosed and treated properly, the tumor may recur or even metastasize; thus, preoperative diagnosis is particularly important for the treatment outcome, and attention should be paid to hepatic cystic lesions with a thickened cyst wall or elevated CA19-9 level. Preoperative needle biopsy is difficult to perform because of the lack of solid tissue in hepatic cystic lesions. Once a malignant tumor is suspected, the most appropriate treatment is complete resection performed in strict accordance with oncological criteria.

## Data Availability

The datasets supporting the conclusions of this article are included in the article.

## Abbreviations

MCN: Mucinous Cystic Neoplasms
CK7: Cytokeratin 7
CK19: Cytokeratin 19
CEA: Carcinoembryonic Antigen
ER: Estrogen Receptor
PR: Progesterone Receptor
CA 19-9: Carbohydrate Atigen19-9
MDT: Multiple Disciplinary Team
WHO: World Health Organisation
